# A mobile microvolume UV/visible light spectrophotometer for the measurement of levofloxacin in saliva

**DOI:** 10.1093/jac/dkaa420

**Published:** 2020-10-22

**Authors:** Jan-Willem C Alffenaar, Erwin M Jongedijk, Claudia A J van Winkel, Margaretha Sariko, Scott K Heysell, Stellah Mpagama, Daan J Touw

**Affiliations:** 1 University of Sydney, Faculty of Medicine and Health, School of Pharmacy, Sydney, Australia; 2 Westmead Hospital, Sydney, Australia; 3 Marie Bashir Institute for Infectious Diseases and Biosecurity, University of Sydney, Sydney, NSW, Australia; 4 University of Groningen, University Medical Center Groningen, Department of Clinical Pharmacy and Pharmacology, Groningen, The Netherlands; 5 Kibong’oto Infectious Diseases Hospital, Kilimanjaro, Tanzania; 6 University of Virginia, Division of Infectious Diseases and International Health, Charlottesville, VA, USA

## Abstract

**Introduction:**

Therapeutic drug monitoring (TDM) for personalized dosing of fluoroquinolones has been recommended to optimize efficacy and reduce acquired drug resistance in the treatment of MDR TB. Therefore, the aim of this study was to develop a simple, low-cost, robust assay for TDM using mobile UV/visible light (UV/VIS) spectrophotometry to quantify levofloxacin in human saliva at the point of care for TB endemic settings.

**Methods:**

All experiments were performed on a mobile UV/VIS spectrophotometer. The levofloxacin concentration was quantified by using the amplitude of the second-order spectrum between 300 and 400 nm of seven calibrators. The concentration of spiked samples was calculated from the spectrum amplitude using linear regression. The method was validated for selectivity, specificity, linearity, accuracy and precision. Drugs frequently co-administered were tested for interference.

**Results:**

The calibration curve was linear over a range of 2.5–50.0 mg/L for levofloxacin, with a correlation coefficient of 0.997. Calculated accuracy ranged from –5.2% to 2.4%. Overall precision ranged from 2.1% to 16.1%. Application of the Savitsky–Golay method reduced the effect of interferents on the quantitation of levofloxacin. Although rifampicin and pyrazinamide showed analytical interference at the lower limit of quantitation of levofloxacin concentrations, this interference had no implication on decisions regarding the levofloxacin dose.

**Conclusions:**

A simple UV/VIS spectrophotometric method to quantify levofloxacin in saliva using a mobile nanophotometer has been validated. This method can be evaluated in programmatic settings to identify patients with low levofloxacin drug exposure to trigger personalized dose adjustment.

## Introduction

TB remains one of the major infectious diseases worldwide, with an estimated number of 10.0 million new cases in 2018, and is the leading killer from a single pathogen.^1^ Driving that mortality is rifampicin-resistant (RR)/MDR-TB, with an estimated 484 000 new patients in 2018.^1^ The multidrug regimen required to treat RR/MDR-TB is less efficacious than that used for drug-susceptible TB. Furthermore, the duration is extended from 9 to as long as 20 months, which represents a burden to both patients and the staff and systems within programmes delivering MDR-TB care.^2^

Moxifloxacin and levofloxacin, the two fluoroquinolones listed as Group A drugs in the WHO consolidated guideline for the treatment of MDR-TB, are the drugs of first choice in combination with bedaquiline and linezolid.^2^ The role of fluoroquinolones is important to prevent acquired resistance in bedaquiline-based shorter all-oral MDR-TB regimens.^3^ Despite being very active drugs, low fluoroquinolone drug exposure is associated with a lower treatment response and acquired drug resistance.^4^ In a large prospective cohort of 832 patients without baseline fluoroquinolone resistance, 11.2% acquired resistance to fluoroquinolones despite good adherence.^5^ Suboptimal moxifloxacin pharmacokinetics may be of particular concern, as only 40% of patients given recommended doses achieve drug concentrations that suppress drug resistance.^6^^,^^7^ Similarly, MDR-TB regimens that give higher doses of fluoroquinolones have been associated with improved outcomes.^8^ Furthermore, pharmacokinetic/pharmacodynamic (PK/PD) studies of moxifloxacin and levofloxacin in pre-clinical models, such as the hollow fibre infection model, have generated clinically achievable PK/PD serum targets that predict bactericidal activity and prevention of acquired resistance.^9^

Considering that PK/PD targets exist for levofloxacin and moxifloxacin, PK variability has been substantial in multiple clinical studies of people being treated for TB,^10^^,^^11^ and higher dosages have been explored to increase drug exposure to improve outcomes;^12^^,^^13^ we therefore argue that fluoroquinolones represent an ideal drug class for therapeutic drug monitoring (TDM) and personalized dose adjustment to optimize the MDR-TB regimen.^2^^,^^14^^,^^15^

Currently, TDM by LC–MS/MS has become the analytical method of choice for quantitation of analytes in biological matrices,^16^ but the use of TDM has been restricted to low TB burden regions with access to personnel, sample shipment procedures and equipment necessary to quantify serum drug exposure.^14^^,^^17^^,^^18^

Although TDM for TB treatment has been recommended for almost two decades,^19^ the financial and logistical challenges of TDM implementation have limited its widespread use.^14^^,^^20^ We and others have previously argued that TDM represents a critical tool in the ‘End TB’ strategies,^18^ especially to limit the amplification and transmission of drug resistance. Treatment should be personalized, and person-centred care can be provided by measuring drug exposure and subsequently individualizing the dose.^21^ While different approaches to implementation may be needed in different settings, the ability to have a semi-quantitative screening test for key drugs such as fluoroquinolones at the community level could then free resources for quantitative measurement of key drugs in selected patients at a regional or central level.^22^Semi-quantitative screening of levofloxacin in saliva to detect patients with unacceptably low or high concentrations seems feasible based on a study comparing plasma and saliva concentrations.^23^

Two alternative matrixes have been explored for the semi-quantitative measurement of drug exposure; oral fluid (saliva) and urine. Although these techniques have their limitations as penetration in oral fluid or renal excretion are prerequisites for these tests to be potentially useful, a major advantage is non-invasive sample collection.^23–29^ As most of the anti-TB drugs including fluoroquinolones have a UV spectrum and are present in the mg/L range, mobile microvolume UV/visible light (VIS) spectrophotometers may be suitable for measuring drug concentrations in saliva and in urine. These devices tend to be user friendly and require a minimum of laboratory skills, which could deliver TDM to a large group of patients that otherwise would not have benefited from traditional TDM programmes.^30^ The aim of this study was therefore to develop a simple, low-cost, robust assay using mobile spectrophotometry to quantify levofloxacin in human saliva that would be applicable for TDM in TB endemic settings.

## Materials and methods

### Materials

Acetaminophen, amoxicillin·3H_2_O, azithromycin, diclofenac sodium, ethambutol diHCl, fluconazole, isoniazid, levofloxacin, linezolid, metformin, sulfamethoxazole and trimethoprim were purchased from Sigma–Aldrich (St Louis, MO, USA). Bedaquiline, ciprofloxacin, dolutegravir, efavirenz and rifampicin were purchased from Alsachim (Illkirch, France). Clofazimine, d-cycloserine, ethionamide and prothionamide were obtained from Toronto Research Chemicals (Ontario, Canada). Pyrazinamide was acquired from Honeywell Fluka (Bucharest, Romania). All reference materials were of ≥98% purity. Ultrapure water (resistivity >15 MΩ·cm at 25°C) was obtained from a Milli-Q Advantage A10 system (Millipore Corporation, Billerica, MA, USA). Absolute methanol of UPLC–MS grade was acquired from Biosolve BV (Valkenswaard, the Netherlands).

Separate stock solutions were used for the preparation of the calibration standards and the quality control (QC) samples. For all experiments, the total volume of (diluted) stock solutions added to filtered drug-free saliva never exceeded 5% (v/v). Calibration standards and QC samples were portioned into vials and stored at −20°C. Vials were discarded after a day of use.

### Equipment and assay procedure

All experiments were performed on a mobile NP80 NanoPhotometer (Implen, München, Germany). The NP80 is a mobile UV/VIS nano spectrophotometer with a scan range of 200–900 nm, a scan time of 2.5–4 s and a bandwidth of <1.8 nm with a sample volume of 0.3–2 μL. Samples of healthy volunteers were collected using a Salivette^®^ (Sarstedt, Nümbrecht, Germany).^31^ Samples were filtered through a Millex-GP (polyethersulphone) of 0.22 μm pore size (Tullagreen, Carrigtwohill, Ireland) using a syringe.^32^ A small drop (≥3 μL) of saliva was placed on the sample surface, with the use of a disposable Pasteur pipette. The path length was set at 0.67 mm and a UV/VIS spectrum was scanned in the 200–900 nm range. The smoothing function was turned off. After each measurement, the sample surface was cleaned, disinfected and dried using lint-free tissues, deionized water and 70% ethanol.

### Method development

According to Lambert–Beer’s law, the light absorbance is directly proportional to the concentration of the absorbing components of the sample.^33^ In our case, this applies to our drug of interest (levofloxacin), but also to all other potentially interfering substances. Finding the wavelength that is most specific for levofloxacin does not make the method impervious to interferences of co-medication or endogenous compounds. Therefore, we developed a strategy to strengthen the selectivity and specificity of spectrophotometry using derivative spectroscopy.^34^ Derivative spectroscopy increases spectral resolution and decreases baseline shifts. Relative broad absorbance bands, caused by light scattering from large molecules (e.g. proteins), are suppressed relative to the sharp absorbance bands of smaller molecules such as levofloxacin. These characteristics allow for detection and quantification of analytes in the presence of a strongly absorbing matrix.^34^^,^^35^

In our described method, the concentration of levofloxacin was evaluated by use of the second-order derivative of the UV/VIS spectrum. As the correlation between the concentration and absorbance of a zero-order spectrum follows Lambert–Beer’s law, we also expect the amplitude of a second-order derivative of the spectrum to exhibit a similar linear function.

$$\frac{d^{2}A}{d\lambda^{2}}=\frac{d^{2}\varepsilon }{d\lambda^{2}}bc$$

where *A* is absorbance, λ is wavelength, ε is extinction coefficient, *b* is sample path length and *c* is sample concentration.

The levofloxacin concentration was quantified by using the amplitude of the second-order spectrum between 300 and 400 nm of seven calibrators. Sample concentrations were calculated from the spectrum amplitude using linear regression. The second-order derivative spectra were calculated by polynomial fitting of the spectral scan, using the Savitsky–Golay method.^36^ Polynomial coefficients were calculated as a vector, using the following matrix equation:^37^

$$a={\left(X^{T}X\right)}^{-1}X^{T}y$$

$$a=\left[\begin{array}{c} a_{0}\\ a_{1}\\ \vdots \\ a_{k} \end{array}\right],X=\left[\begin{array}{ccccc} 1 & x_{1} & x_{1}^{2} & \ldots & x_{1}^{k}\\ 1 & x_{2} & x_{2}^{2} & \ldots & x_{2}^{k}\\ \vdots & \vdots & \vdots & \ddots & \vdots \\ 1 & x_{n} & x_{n}^{2} & \ldots & x_{n}^{k} \end{array}\right],y=\left[\begin{array}{c} y_{1}\\ y_{2}\\ \vdots \\ y_{n} \end{array}\right],$$

where *k* is polynomial order and *n* is wavelength interval.

The second-order derivative of the polynomial was expressed by:

$$\frac{d^{2}y}{dx^{2}}=2a_{2}+6a_{3}x+12a_{4}x^{2}+\ldots +(k^{2}-k)a_{k}x^{k-2}$$

The wavelength interval and polynomial order of the polynomial fitting were optimized for deconvolution and signal-to-noise ratio, by minimization of the bias and precision of calculated levofloxacin concentration in the presence of various potential interferents. All calculations were done by importing all raw data into a proprietary Excel spreadsheet (Microsoft, Redmond, WA, USA).

### Method validation

Method validation was performed according to FDA and EMA guidelines for selectivity, specificity, linearity, accuracy and precision.

The levofloxacin calibration curve consisted of seven points at the concentrations of 2.50, 5.0, 10.0, 20.0, 30.0, 40.0 and 50.0 mg/L, which is suitable for clinical practice as levofloxacin peak concentration ranges from 8 to 40 mg/L.^38^ The lower limit of quantitation (LLOQ), low, medium and high QC concentrations were at 2.50, 5.00, 25.0 and 40.0 mg/L, respectively. For specificity, six human drug-free saliva samples, each obtained from separate healthy volunteers, were tested for interference. Measurements of these drug-free samples ideally result in a levofloxacin concentration less than the LLOQ. For selectivity, these drug-free samples were spiked with levofloxacin at the LLOQ concentration. Measurements of the spiked samples ideally result in a bias <20%. Interpatient variance was assessed by spiking separate drug-free saliva samples from six different healthy volunteers at low and high concentrations. Bias and precision should be <15% at all concentrations. To assess the effect of exogenous components (e.g. other medicines), a pool of single donor, drug-free saliva was spiked at the LLOQ and high levofloxacin concentrations. The unspiked drug-free saliva and the spiked saliva were additionally spiked with medicines likely to be present in our patient population. The drug-free saliva was spiked at the expected maximum concentration (*C*_max_) of these drugs in saliva retrieved from the literature. If a *C*_max_ value in saliva could not be retrieved from the literature, *C*_max_ in plasma was used instead.^19^^,^^24^ All spiked samples were analysed in five replicates on a single day. Unspiked blank saliva ideally result in responses less than the LLOQ. Drug-free saliva samples spiked with levofloxacin ideally result in a bias <20% at the LLOQ and a bias <15% at high concentration. Accuracy and precision were determined by measuring the LLOQ, low, medium and high QC samples in replicates of five over three separate days. The samples were quantified using a single seven-point calibration curve that was measured on that same day. Within-day, between-day and overall precision were calculated with the use of a one-way ANOVA. The acceptance criterion for bias and precision was <20% at the LLOQ and <15% at the low, medium and high concentrations.

## Results

**Figure 1.**
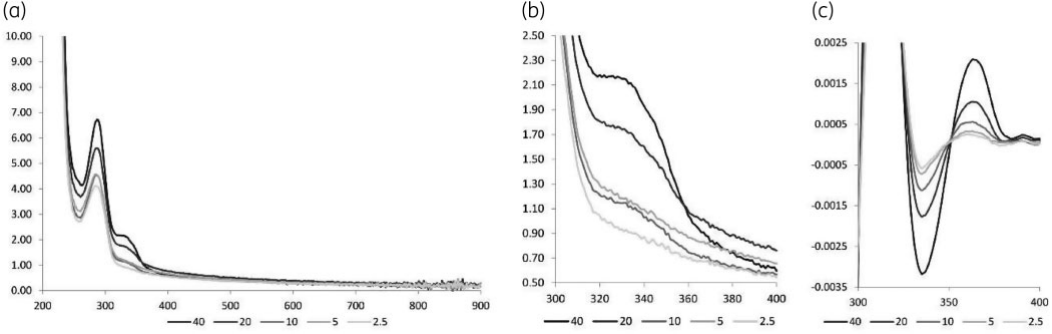
Spectra of levofloxacin in saliva. (a) Full zero-order spectra of levofloxacin in saliva at 2.5, 5, 10, 20 and 40 mg/L, (b) detail of the zero-order spectra and (c) detail of second-order spectra [S-G(8,61)].

Absorbance scans of saliva samples showed clear baseline shifts. Figure 1(a and b) shows scans of five concentrations of levofloxacin spiked to the same drug-free saliva, doubling the levofloxacin concentration at every successive concentration. Theoretically the absorbance at 285 nm and 320 nm can be used to quantify levofloxacin, according to Lambert–Beer’s law. However, the baseline shifts, from sample to sample, resulted in a lack of correlation between the levofloxacin concentration and the absorbance. Figure 1c shows that the amplitudes of the second-order derivative of the same spectra do correlate with the levofloxacin concentration. In effect, the concavity of the inflection point of the zero-order absorbance band is used to quantify levofloxacin in the saliva sample.

**Figure 2.**
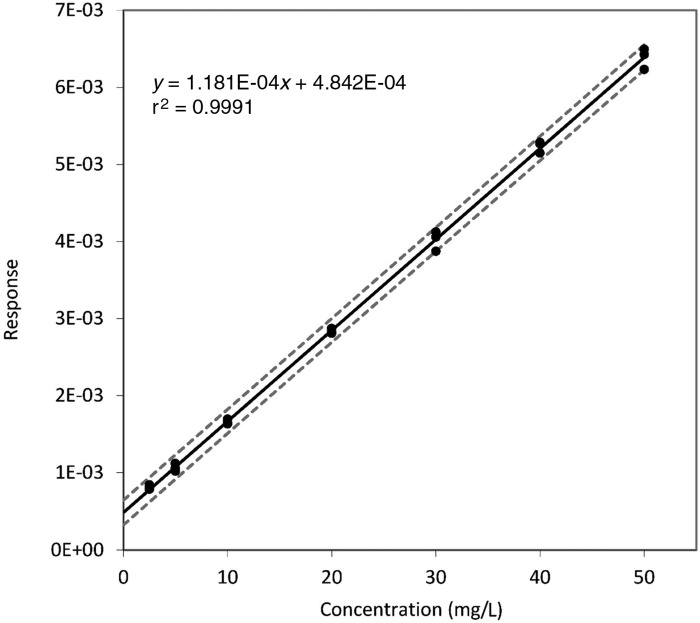
Calibration curve in drug-free saliva (*n *=* *3) with 95% CI.

Specificity and selectivity were assessed by analysing six separate drug-free samples. All six drug-free samples resulted in responses below the response of the LLOQ. Biases ranged from 87% to 115% at the LLOQ level, from 93% to 113% at the low concentration and from 94% to 102% at the high concentration. Precision was 10.4%, 7.1% and 2.9%, respectively. Linearity was assessed using a seven-point calibration curve (*n *=* *3). The linear range was proven to be 2.5–50 mg/L, with a weighting factor of 1 (*r*^2^=0.9991, *n *=* *3, Figure 2). The accuracy, within-day precision, between-day precision and overall precision were assessed at four concentrations. The results are shown in Table 1.

**Table 1.** Accuracy and precision

	Value at different concentrations			
Criterion	LLOQ	Low	Medium	High
Nominal concentration (mg/L)	2.50	5.00	25.0	40.0
Accuracy [bias (%)]	−5.2	0.4	1.9	2.4
Within-day precision [CV (%)]	11.4	4.4	1.0	0.7
Between-day precision [CV (%)]	11.4	7.8	1.9	2.0
Overall precision [CV (%)]	16.1	9.0	2.1	2.1

CV=coefficient of variation calculated as (SD/mean) × 100%.

**Figure 3.**
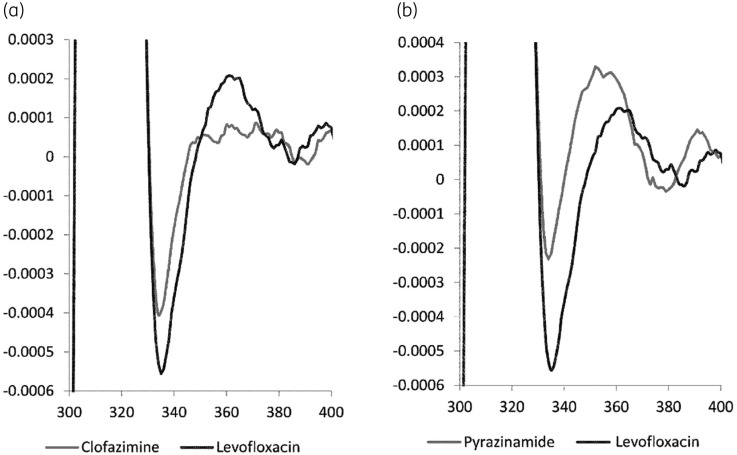
Differences in second-order derivative spectra of different co-administered drugs. (a) Clofazimine at a concentration of 0.4 mg/L gives a lower response than the response of the levofloxacin LLOQ and (b) pyrazinamide at a concentration of 42 mg/L gives a higher response than the response of the levofloxacin LLOQ.

The effect of co-medication on the quantitation of levofloxacin was minimized by optimizing the Savitsky–Golay method. Figures S1 and S2 (available as Supplementary data at *JAC* Online) show how the calculation of the second-order derivative spectrum at the LLOQ, by the Savitsky–Golay method, is affected by changes in the wavelength interval and order of the polynomial fit. Of all tested combinations of polynomial order and wavelength interval, a polynomial of the eighth order fitted to a 61 nm interval [S-G(8,61)] gave the best overall results. Figure 3 shows the differences in second-order derivative spectra between drug-free saliva spiked with 0.4 mg/L clofazimine, drug-free saliva spiked with 42 mg/L pyrazinamide and drug-free saliva spiked with levofloxacin at the LLOQ. All drug-free saliva samples spiked with potential co-medication (Table 2) gave responses of <2.5 mg/L levofloxacin in the absence of levofloxacin, with the exception of rifampicin and pyrazinamide. Rifampicin and pyrazinamide resulted in a positive bias of 171.7% and 27.3% of levofloxacin at the LLOQ concentration, respectively. This means that a levofloxacin concentration is reported as 3.2 mg/L instead of 2.5 mg/L in the presence of a pyrazinamide concentration of 42 mg/L. This difference will not affect clinical decision making as the absolute level is very low as levofloxacin peak concentrations typically range from 8 to 40 mg/L.^38^

**Table 2.** Effect of co-medication and anti-TB drugs on levofloxacin results

Drug	Tested concentration (mg/L)	Bias (CV) of LLOQ (2.5 mg/L) (%)	Bias (CV) of high (40 mg/L) (%)
Acetaminophen	12.0	2.1 (4.7)	1.9 (0.7)
Amoxicillin	6.5	6.0 (6.4)	6.9 (0.9)
Azithromycin	0.6	8.1 (6.3)	5.0 (0.9)
Ciprofloxacin	0.4	9.0 (3.6)	6.4 (0.6)
Diclofenac	1.5	4.8 (6.1)	1.0 (1.3)
Dolutegravir	1.0	−11.1 (2.5)	−2.1 (2.4)
Efavirenz	1.0	−1.6 (3.9)	−1.3 (1.6)
Fluconazole	10.0	7.7 (4.6)	0.1 (0.5)
Metformin	2.0	−9.4 (8.0)	−3.2 (1.8)
Sulfamethoxazole	9.0	6.5 (3.4)	0.7 (0.7)
Trimethoprim	4.5	4.2 (3.7)	0.6 (0.8)
Bedaquiline	3.5	11.8 (5.7)	2.3 (0.6)
Clofazimine	0.4	6.3 (6.0)	4.1 (0.6)
Cycloserine	19.5	0.3 (2.9)	4.1 (0.8)
Ethambutol	1.3	8.6 (10.5)	1.6 (0.7)
Ethionamide	2.5	7.6 (6.8)	8.4 (0.8)
Isoniazid	7.5	12.2 (4.1)	2.4 (1.2)
Linezolid	10.0	8.7 (7.8)	3.0 (1.5)
Prothionamide	5.0	1.9 (4.8)	3.7 (0.8)
Pyrazinamide	42.0	27.3 (2.3)	9.3 (0.8)
Rifampicin	12.0	171.7 (2.0)	21.0 (0.6)

CV=coefficient of variation calculated as (SD/mean) × 100%.

## Discussion

We developed an accurate and precise analytical method suitable for the measurement of levofloxacin in human saliva using a mobile microvolume UV/VIS spectrophotometer. The main challenge during the development of this method was ensuring acceptable selectivity, specificity and robustness in the presence of co-medication. Because we aimed at an easy-to-use assay under field conditions, it was decided that extensive sample clean-up was not acceptable. After exploring different strategies for isolating the response of levofloxacin from various background signals, such as the subtraction of a drug-free saliva spectrum or standard addition per sample, it became apparent that derivative spectroscopy was the most viable option for routine use.

Derivative spectroscopy requires complex mathematics to generate reproducible results. As such, considerable effort was dedicated to the development of pre-specified calculations to make concentration determination virtually effortless during routine use. Ideally, the Savitsky–Golay method should be integrated into the firmware of the mobile UV/VIS spectrophotometer. The Savitsky–Golay method ensures optimal robustness in the presence of co-medication when a 61 nm range was used to fit an eighth-order polynomial. Nevertheless, it must be commented that the presence of rifampicin and pyrazinamide can affect the measurement of levofloxacin at the LLOQ. Given that levofloxacin is used primarily as a core agent against MDR-TB, which is by definition resistant to rifampicin, it will be highly unlikely that rifampicin will be present in our intended patient group. The updated WHO guidelines for MDR-TB advises a regimen with at least five effective anti-TB drugs during the intensive phase.^2^ Currently, pyrazinamide is listed as a Group C drug and only to be counted as an effective drug in cases where susceptibility has been proven by drug susceptibility testing.^2^ Therefore, the use of pyrazinamide in our intended patient group is possible, but becoming less common in current global MDR-TB strategies. Moreover, our validation showed that the level of pyrazinamide interference is negligible at higher levofloxacin concentrations. Furthermore, as samples are collected after the absorption phase to capture the peak concentration, the interference of pyrazinamide is not expected to have clinical implications. Developed limited sampling strategies have shown that single or multiple samples collected after drug administration can be used to quantify levofloxacin exposure, which mitigates the risk of interference.^39^

To demonstrate the usefulness of this method, as a next step, we will perform a clinical validation in an MDR-TB endemic setting among people being treated with levofloxacin and pyrazinamide utilizing paired saliva and plasma collection. Saliva samples will be measured not only using the UV/VIS spectrophotometer but also using LC–MS/MS^31^ to show if other factors potentially impact the results obtained with the nanophotometer. In our opinion, the use of the mobile nanophotometer has the potential to comply with most criteria defined for diagnostics tests in low resource settings [ASSURED (Affordable, Sensitive, Specific, User-friendly, Rapid and robust, Equipment-free and Deliverable to end-users)].^40^ Compared with traditional chromatographic methods for TDM, the nanophotometer is more affordable. At least for levofloxacin, we have shown that the assay is sensitive and specific for its purpose. The simple sample preparation required for the assay ensures user-friendliness and a high degree of acceptance with end-users. UV/VIS spectrometry is fast and robust, but requires equipment. Fortunately, the equipment can be used in field conditions, which means that samples of patients do not have to be transported to a laboratory and results are immediately available for the end-users. For implementation in routine care, we envisage that the levofloxacin saliva AUC can be adequately estimated using a limited sampling strategy in combination with linear regression Bayesian dose selection^39^ and converted into a plasma AUC based on the saliva/plasma penetration ratio. Subsequently, the required dose to target the appropriate AUC to achieve an AUC/MIC ratio associated with optimal kill^9^ can be calculated. The new dose can be selected on available tablet size rounded up to the closest whole tablet up to a maximum of 25 mg/kg daily^9^ while adequately monitoring patient safety.^41^

To conclude, we have developed and validated a UV/VIS spectrophotometric assay for measurement of levofloxacin concentration in saliva. After clinical validation, this assay will greatly expand access to personalized dosing strategies for people with MDR-TB at a community level.

## Funding

This project was financially support by the Bill & Melinda Gates Foundation, Grant Challenges programme (grant number OPP1191221).

## Transparency declarations

None to declare.

## Supplementary data

Figures S1 and S2 are available as Supplementary data at *JAC* Online.

## Supplementary Material

dkaa420_Supplementary_Data
