# Supplementary material for: A mobile microvolume UV/visible light spectrophotometer for the measurement of levofloxacin in saliva
Source: J Antimicrob Chemother. 2020 Oct 22;76(2):423–9. doi: 10.1093/jac/dkaa420 (PMC7816168; doi:10.1093/jac/dkaa420)
Supplement: dkaa420_Supplementary_Data [file dkaa420_supplementary_data.docx]

**Supplementary data**


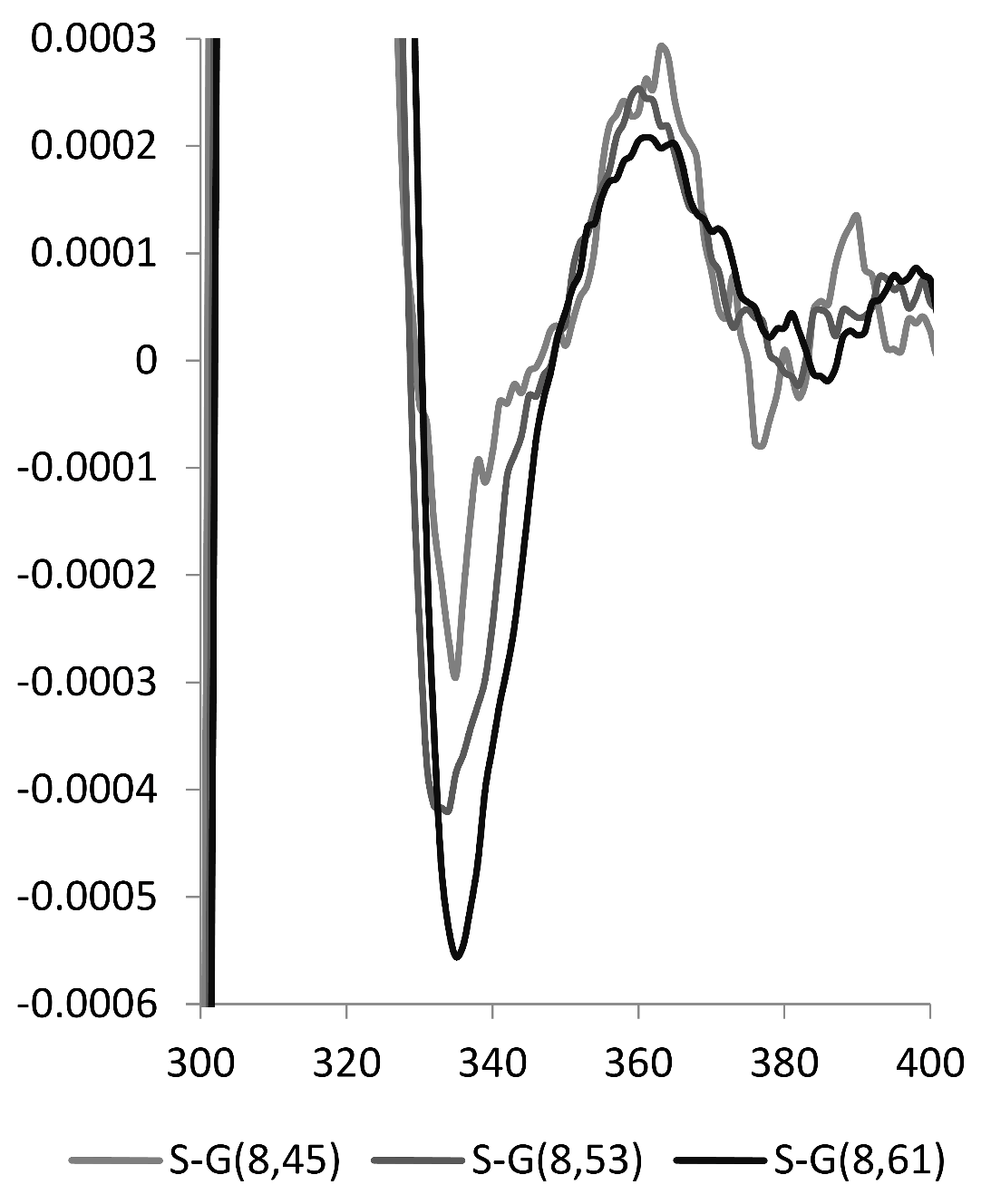


**Figure S1.** The effect of different intervals of polynomial fit on spectral noise.


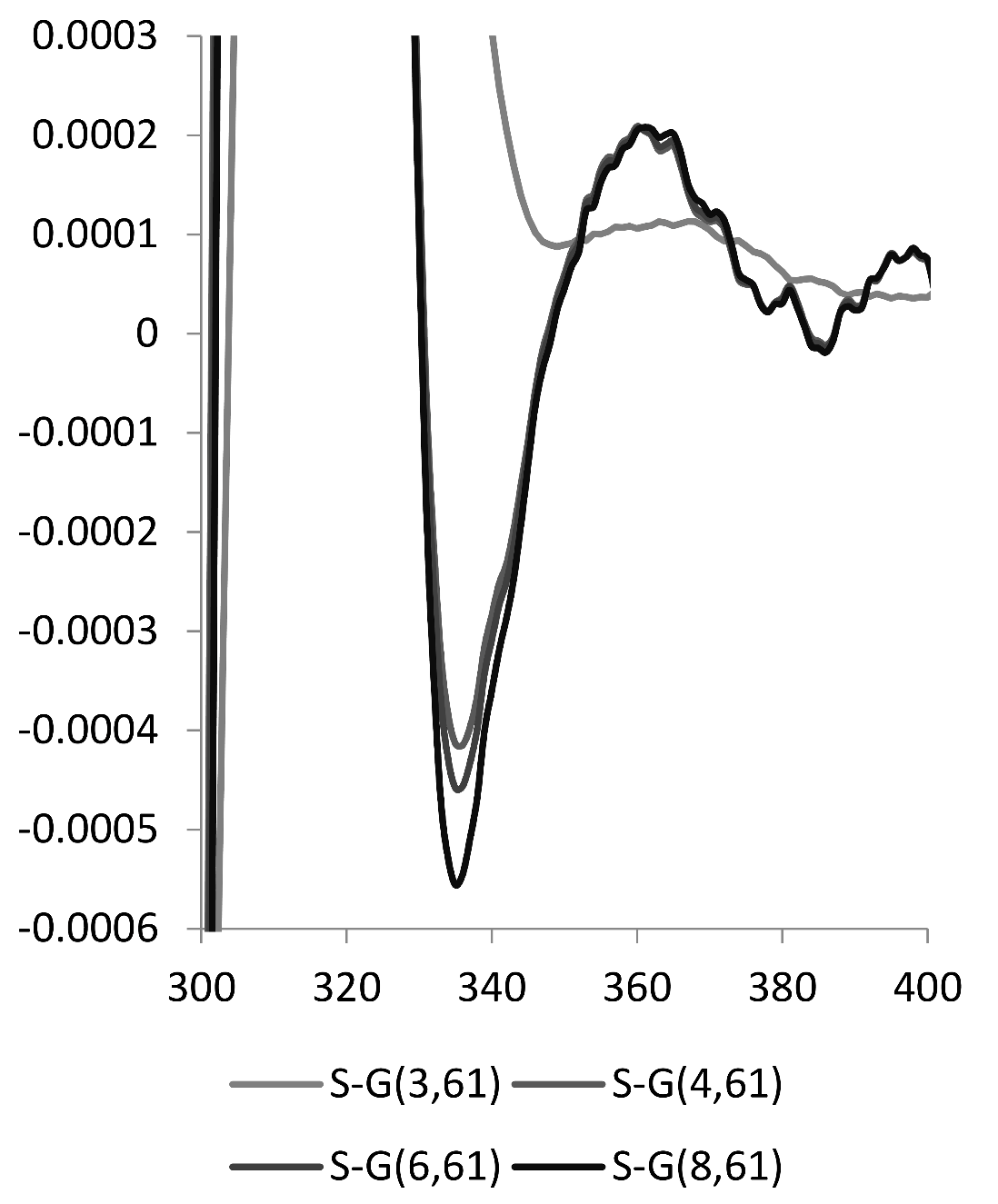


**Figure S2.** The effect of different polynomial orders on the response.
